# Quantifying effects of solar power adoption on CO_2_ emissions reduction

**DOI:** 10.1126/sciadv.adq5660

**Published:** 2025-07-30

**Authors:** Arpita Biswas, Minghao Qiu, Danielle Braun, Francesca Dominici, Daniel Mork

**Affiliations:** ^1^ Department of Computer Science, Rutgers University, New Brunswick, NJ, USA.; ^2^ School of Marine and Atmospheric Sciences, Stony Brook University, Stony Brook, NY, USA.; ^3^ Program in Public Health, Stony Brook University, Stony Brook, NY, USA.; ^4^ Department of Biostatistics, Harvard T.H. Chan School of Public Health, Boston, MA, USA.; ^5^ Department of Data Science, Dana-Farber Cancer Institute, Boston, MA, USA.

## Abstract

We quantify the effect of solar power adoption in reducing carbon dioxide (CO_2_) emissions from the US electricity sector using 5 years of Energy Information Administration data, starting 1 July 2018. By tailoring a distributed lag statistical model, we estimate the immediate and time-lagged effects of increased solar generation on reducing CO_2_ emissions within a region. Our analysis highlights how solar adoption in one region affects CO_2_ emissions in neighboring regions, emphasizing the potential for collaborative efforts. We estimate that a 15% increase in solar generation is associated with an annual reduction of 8.54 million metric tons (MMT) of CO_2_ emissions, contributing 12.38% toward the yearly target of 69 MMT CO_2_ reductions needed to cut 1380 MMT of CO_2_ in 20 years, as per the Environmental Protection Agency rule on fossil fuel power plants. This study offers insights for policymakers and stakeholders in achieving CO_2_ reduction targets through increased solar generation.

## Teaser

Analyzing the impact of solar power adoption on CO_2_ reduction targets provides key insights for policymakers and stakeholders.

## INTRODUCTION

In the United States, electricity is generated from a diverse mix of sources, such as coal, natural gas, nuclear, petroleum, hydro, solar, and wind, and the dependence on these sources varies between different geographical regions. On the basis of data from the US Energy Information Administration (EIA), 60% of the electricity generation in the United States in 2023 relied on fossil fuel–fired power plants (coal, natural gas, petroleum, and other gases). In contrast, solar energy contributed only to 3.9% of total electricity generation (*1*); fig. S1 shows the contributions of the various energy sources in each geographical region of the United States, averaged over a period of 5 years starting 1 July 2018. We observed that electricity generation in the California and Northwest regions is highly dependent on renewable energy sources such as solar and hydroelectric power because these regions have abundant sunlight and water resources, while New England is highly dependent on natural gas and nuclear power to meet its electricity demands. The South depends mainly on a combination of coal, natural gas, and nuclear energy, and coal plays a more prominent role in the central and Midwest regions.

The heavy dependence on fossil fuel power plants that burn coal, petroleum, and natural gas for electricity generation poses two challenges. First, these power sources have been an important contributor to air pollutant emissions (*2*–*4*), resulting in higher levels of fine particulate matter (PM_2.5_) and rates of mortality and morbidity (*5*–*8*). Second, fossil fuel power plants burning coal, natural gas, and petroleum fuels represented 99% of 1.65 billion metric tons of CO_2_ emissions associated with the generation of utility-scale electric power in the United States in 2022 (*9*, *10*), implying that these power plants play a central role in CO_2_ emissions. The large role of fossil fuel power plants in the emission of CO_2_ and ambient levels of PM_2.5_ underscore the need to regulate these power sources. On 25 April 2024, the US Environmental Protection Agency (EPA) announced regulations to effectively eliminate CO_2_ emissions from the US electricity sector (*11*). The proposed measures include various advanced technologies, such as combined cycle systems, ultra-supercritical boilers, and combined heat and power systems, and are expected to reduce CO_2_ emissions by 1.38 billion metric tons between 2028 and 2042. However, only implementing these end-of-pipe technologies may not be sufficient to completely eliminate CO_2_ emissions from the electricity sector by 2050, so we need to investigate the adoption of other sources of energy, such as solar energy, and gradually reduce the dependence on fossil fuel power plants. Toward this end, the International Energy Agency (IEA) emphasized the need to scale renewables, such as solar and wind, to achieve net zero emissions by 2050 (*12*). In this study, we investigate the extent to which increases in solar power generation lead to reductions in CO_2_ emissions from the US electricity sector.

Many studies have documented that the deployment of renewable energy can reduce greenhouse gas and air pollution emissions, leading to improved air quality and related human health outcomes (*13*–*16*). A commonly used approach to estimate the effect of increased solar power generation on CO_2_ emission reductions is known as “primary energy equivalent” (*17*). It involves multiplying the additional solar generation by the region-specific CO_2_ emission factor for the generation of electricity from fossil fuels during that year. This method fails in low–fossil fuel generation regions because of the assumption that, under constant electricity demand, any increase in solar power generation directly displaces the equivalent amount of electricity that would have been generated by fossil fuel power plants. Therefore, a number of sophisticated statistical methods have been proposed. For example, regional marginal emission rates are determined by calculating the slope of a best-fit line between generation and emissions within a region (*18*), with improvements to account for temporal variations (*13*). Another method, called EnergyPLAN (*19*), solves an optimization problem to minimize fossil fuel use subject to the constraint that balances demand and supply from the electricity system from all sources each hour. In contrast, the AVERT tool (*20*, *21*) uses a data-driven method with hour generation and emissions data to analyze which energy-generating units respond to increases in renewable power generation and then replicates the changes using Monte Carlo simulations. Although numerous other methods (*22*, *23*) exist that we are unable to exhaustively discuss here, none of the existing solutions account for the time-lagged effects—specifically, how increased solar power generation in a given hour influences delayed changes in CO_2_ emissions over subsequent hours.

Accurately quantifying the effects of solar power on CO_2_ emissions still faces several challenges. First, owing to geographical variations in solar resources and the electricity grid, the effects of solar power on CO_2_ emissions can be very different between regions. Second, electrical energy can be exported from one region to a nearby region, and therefore, changes in renewable energy generation, such as solar power, could also affect CO_2_ emissions in a neighboring region (*24*, *25*), requiring the quantification of interregional effects. Third, increasing solar generation may lead to a potential delayed decrease in CO_2_ emissions over the following hours within the same region or in a neighboring region that imports energy; therefore, time-lagged effects need to be quantified.

To address these challenges, we constructed a statistical model that estimates the association between the possible cumulative decrease in hourly CO_2_ emissions within each geographic region and the increase in solar generation for a given hour and each of the previous 12 hours (*26*–*28*). We fit separate models for each region to account for their varied electricity demand and solar-CO_2_ associations. Furthermore, we consider a time-lagged relationship between increases in solar generation and changes in CO_2_ emissions in neighboring export regions.

We created a publicly available data pipeline that integrates data from different data sources. More specifically, we analyze hourly electricity generation from solar power, hourly electricity demand, electric energy flow (exports), and CO_2_ emissions from US electricity grids for 13 distinct regions—California, Carolinas, Central, Florida, Mid-Atlantic, Midwest, New England, New York, Northwest, Southeast, Southwest, Tennessee, and Texas—between 1 July 2018 and 30 June 2023, as provided by EIA (*29*). Using these datasets, we address the following scientific questions: (i) if we were to increase the hourly solar generation in a given region by 5, 10, 15, or 20%, what would be the resulting change in CO_2_ emissions within the same region? (ii) If we were to increase the hourly solar generation in a given region by 5, 10, 15, or 20%, what would be the resulting changes in CO_2_ emissions in its neighboring regions?

We focus on the change in CO_2_ emissions associated with increase in solar generation, driven by the expansion of solar generating capacity of a region. Solar generating capacity [measured in megawatts (MW)] is the maximum amount of electricity that can be produced during peak solar periods. The solar generating capacity can be increased by various means, for example, by increasing the number of solar panels or by increasing the efficiency of existing solar panels. Increasing solar generating capacity in a region implies that solar power generation per hour (in megawatt-hours) could be increased. Throughout the paper, when referring to an “increase in solar power generation,” we assume that the solar generating capacity of the corresponding region has expanded accordingly to support the observed increase in generation.

## RESULTS

In recent years, there has been a noticeable increase in solar generating capacity across the United States. For example, in California, solar generating capacity has increased by approximately 15% from 16,317.75 MW in 2021 to 18,610.18 MW in 2022. We observed that the increase in solar generating capacity corresponds to a proportional percentage increase in hourly solar power generation throughout the day. Throughout this study, we consider increases in hourly solar power generation with respect to its median hourly value in 2022 (because 2022 was the most recent year for which we had complete annual data). For clarity, let us look at the median solar power generation per hour during a few selected timeframes in California in 2022. At 0400, 0800, 1200, 1600, and 2000 hours, the median solar power generation was approximately 0, 5403, 12,563, 11,249, and 53 megawatt-hours (MWh), respectively. A 15% increase in solar power generation would translate into increased solar power generation during these hours equating to 0, 6213, 14,447, 12,936, and 61 MWh, respectively. Because the achievable increases in solar power generation are different for different regions, we subsequently assumed a 5, 10, 15, or 20% increase in the hourly solar power for each region and then estimate the associated change in CO_2_ emissions corresponding to the increases in solar power generation.

**Fig. 1.**
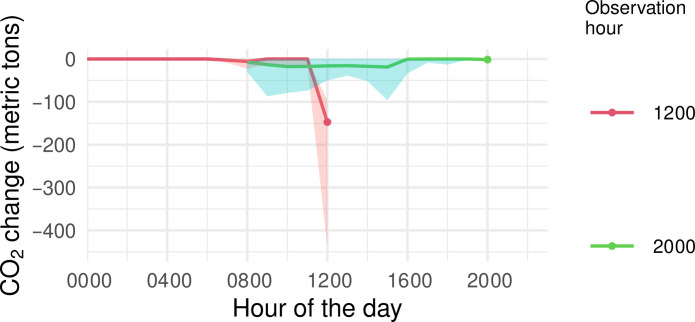
Estimated changes in CO_2_ emissions during two specific time points in California, at noon (marked in red) and 2000 (marked in light blue), associated with a 15% increase in solar power generation in the same hour (immediate effect) and during each of the preceding 12 hours (time-lagged effect). For instance, the light blue value corresponding to x-label 1200 indicates that a 15% increase in solar power generation at 1200 is associated with a 16.08–metric ton change in CO_2_ emissions at 2000. The shaded areas along the color-coded trajectories denote 95% credible intervals.

We first estimate the effect of increasing the hourly solar power generation by 15% on the potential reduction in CO_2_ emissions within the same region. For illustration purposes, we focus on California and the estimated changes in CO_2_ for two specific time points (1200 and 2000). Figure 1 shows the estimated change in CO_2_ emissions during those two time points (color coded as red and light blue, respectively), associated with increases in solar in each of the last 12 hours. We found that a 15% increase in solar power at noon is associated with a reduction of 147.18 (95% CI: 96.62, 438.02) metric tons of CO_2_ during that hour (refer to Fig. 1, denoted by the red line at *x*-axis value 1200). Increasing solar power can also reduce CO_2_ emissions in the grid with a temporal delay. For example, a 15% increase in solar power at noon is also associated with a reduction of CO_2_ at 2000 by 16.08 (95% CI: 0.00, 50.13) metric tons (refer to Fig. 1, denoted by the light blue line at *x*-axis value 1200). These findings highlight the intricate relationship between hourly solar power and its impact on CO_2_ reductions, both immediate and delayed. In addition, we observed a decreased immediate change in CO_2_ emissions during the early morning and postsunset periods. This phenomenon can be attributed to the inherently lower generation of solar power during these times. Note that the median solar values during nighttime hours (from 2200 to midnight through 0600) are 0 MWh. Consequently, a 15% increase in hourly solar power over the median maintains a static increase of 0 MWh during nighttime hours, resulting in zero changes in CO_2_ emissions during these hours.

**Fig. 2.**
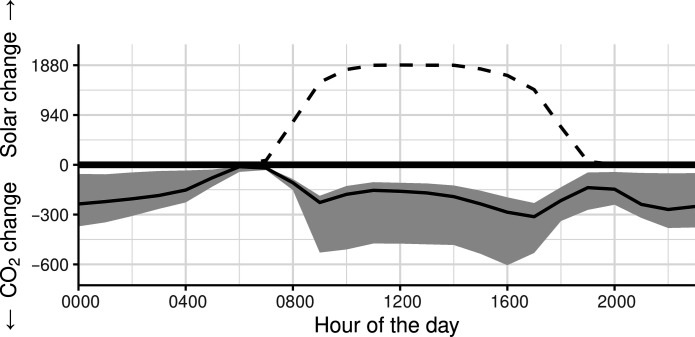
The positive *y* axis shows a 15% increase in solar power generation (in megawatt-hours) at each hour and the negative *y* axis shows the estimated cumulative change in CO_2_ emissions (metric tons) at each hour, summing over the changes in CO_2_ due to the increase in hourly solar generation during the past 12 hours.

Figure 2 shows the cumulative reduction in hourly CO_2_ emissions associated with a 15% increase in solar power at each hour during the past 12 hours. For example, we found that a 15% increase in solar power during each hour from 0800 to 2000 is associated with a cumulative reduction of 147.04 (43.30, 240.60) metric tons of CO_2_ emissions at 2000 (as shown on the negative *y* axis at the value of the *x*-axis 2000 in Fig. 2).

**Fig. 3.**
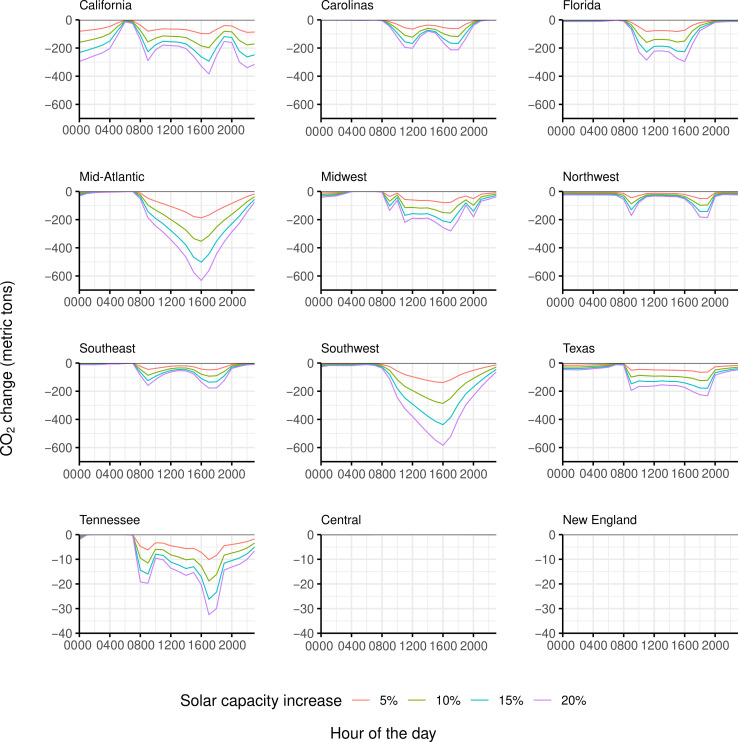
Estimated cumulative hourly change in CO_2_ emissions in each region for a 5, 10, 15, and 20% increase in the solar power during each of the past 12 hours within that region. Because the change in CO_2_ emissions is very low for Central, Tennessee, and New England, we adjusted the *y*-axis scale for those regions to enhance plot visibility. However, for New England, the CO_2_ reductions are consistently 0, resulting in no visible data on the plot.

Figure 3 shows the cumulative reductions in CO_2_ at each hour of the day corresponding to a 5, 10, 15, and 20% increase in hourly solar power within the same region during each of the last 12 hours. For example, the light blue line for California in Fig. 3 represents the cumulative CO_2_ changes associated with a 15% increase in solar power generation, as seen in the negative axis of Fig. 2. In general, we found that regions such as California, Florida, the Mid-Atlantic, Midwest, Southwest, and Texas show a prominent reduction in CO_2_, with hourly changes of more than 200 metric tons during some hours of the day. In contrast, for Tennessee, Central, and New England, the estimated cumulative reduction in CO_2_ emissions is minimal (32.46, 0.06, and 0, respectively) even in the scenario of a 20% increase in solar power generation.

**Fig. 4.**
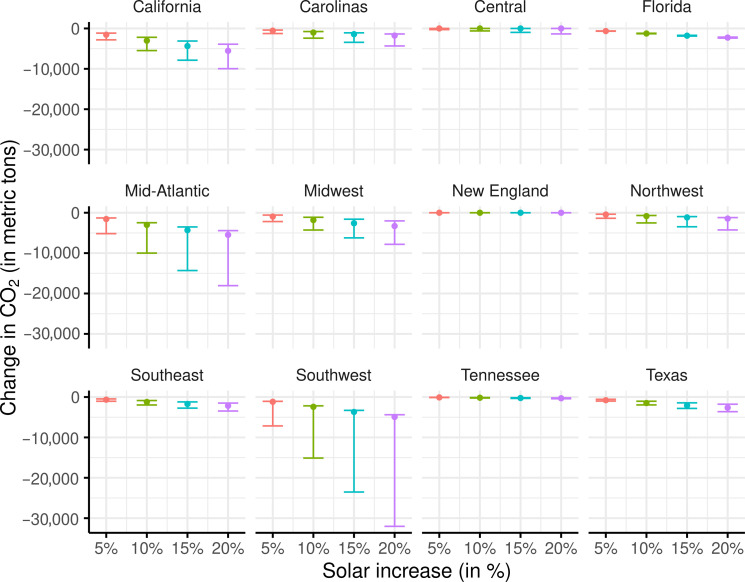
Estimated total change in CO_2_ (95% CI) per day associated with 5, 10, 15, and 20% increase (colors) in solar generation during each of the 24 hours in each region. Regional variations are substantial, with regions like the Mid-Atlantic, California, Southwest, Midwest, and Texas exhibiting a stronger association of increasing solar with CO_2_ emission reduction compared to regions such as New England, Central, and Tennessee.

Figure 4 shows the estimated cumulative change in CO_2_ emissions per day (along with the credible interval 95%) in various regions for a 5, 10, 15, and 20% increase in hourly solar power generation at each hour of the day. These estimates are computed using the sum of potential hourly changes in CO_2_ over all 24 hours, as shown in Fig. 3. In California, we found that a total daily reduction of 4357 metric tons of CO_2_ emissions is associated with a 15% increase in solar power at each hour of the day. Table S1 shows the estimated daily CO_2_ reductions for each region, corresponding to an increase of 5, 10, 15, and 20% in solar power generation.

**Fig. 5.**
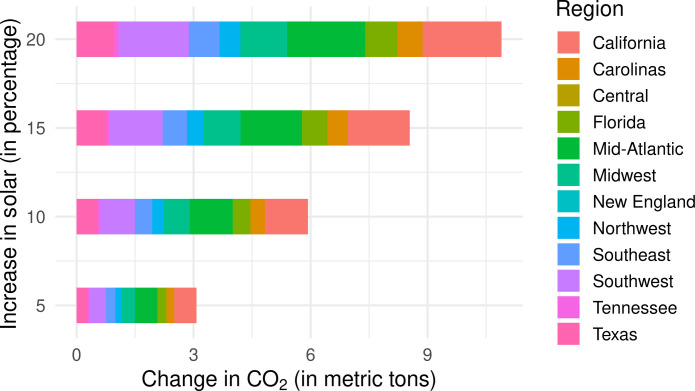
The estimated annual change in CO_2_ emissions (*x* axis) in each region (colors), associated with increased solar generation at every hour of the day within each region and also in the neighboring regions. Each horizontal bar corresponds to a specific percentage increase in solar generation (5, 10, 15, and 20%). Within each horizontal bar, the colored segments represent the estimated CO_2_ reductions in different US regions (such as California, Carolinas, Central, etc.).

These per-day estimates are then used for computing the estimated change in CO_2_ emissions in a year upon increasing solar generation, assuming that other conditions remain constant. Under the assumption that we increase solar power generation by 15% for each hour of the day throughout a year, Fig. 5 shows a cumulative reduction of CO_2_ emissions by 1.59 million metric tons (MMT) in California. Similarly, in Southwest, under the assumption that we increase solar power generation by 15% at each hour throughout the year, we estimate a cumulative decrease of about 1.35 MMT of CO_2_. Summing across all 12 regions, a 15% increase in solar power generation leads to an estimated annual reduction of 8.54 MMT of CO_2_ emissions, accounting for 12.38% of the yearly target of 69 MMT of CO_2_ reductions needed to achieve the EPA’s goal of reducing 1380 MMT of CO_2_ emissions in the next 20 years. These policy implications underscore the importance of promoting solar power adoption and expansion for a cleaner and more sustainable future.

**Fig. 6.**
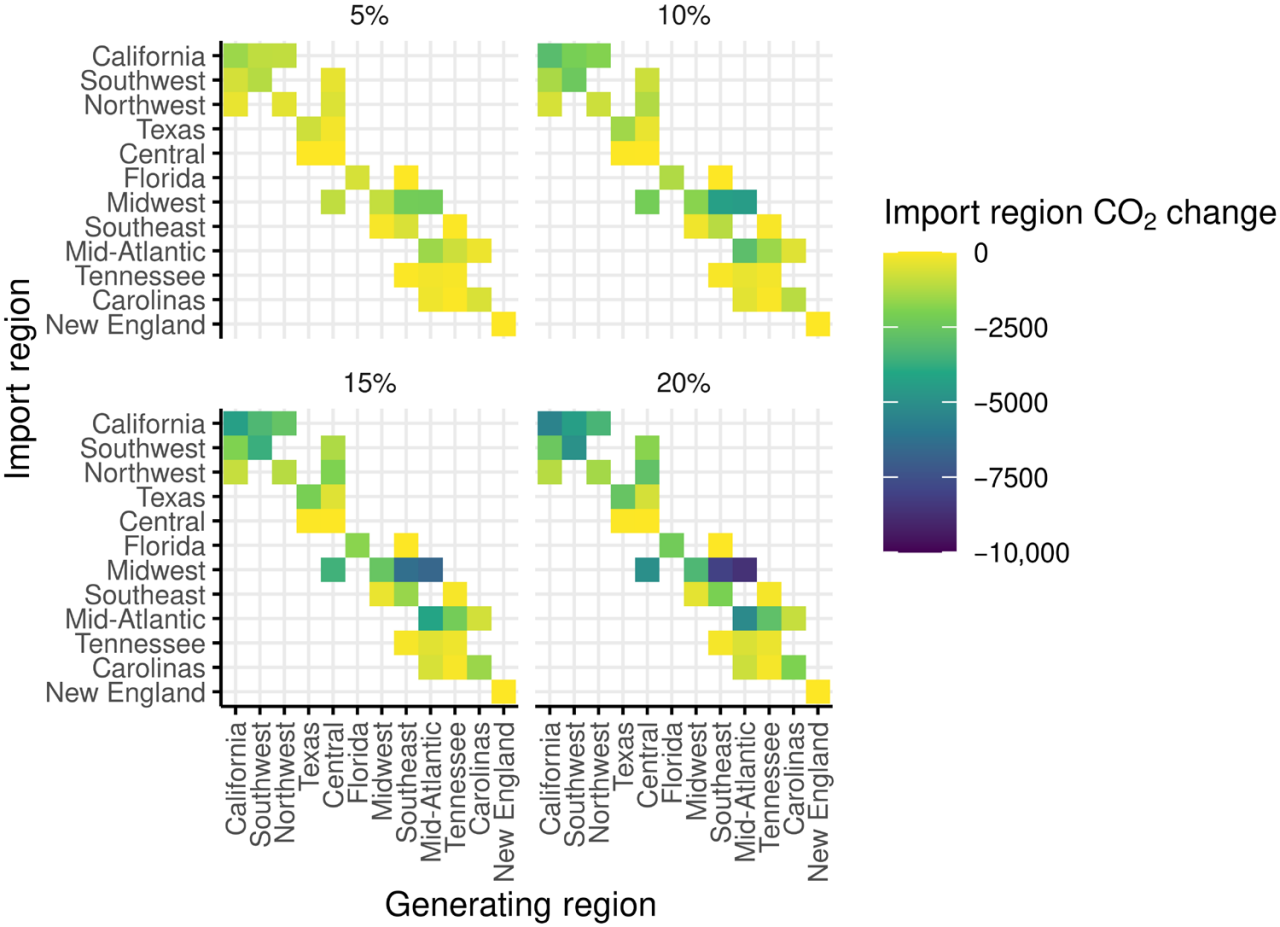
A heatmap of the total change in CO_2_ (metric tons) per day in the import region corresponding to an increase of 5, 10, 15, or 20% in solar power generation in the generating region. Note that our analysis focuses only on pairs of regions with nonzero import/export values, highlighted by colored squares.

Last, to gain insight into the interregional implications of increasing solar power generation, we study the effect of the change in CO_2_ emissions in a region associated with an increase in solar power generation in each of its neighboring regions. Our analysis shows that there are several regions for which increasing solar power has an impact on the CO_2_ reduction in neighboring regions. For example, we estimate that a 15% increase in solar capacity in California is associated with a total reduction of 913 and 1942 metric tons of CO_2_ emissions per day in the Northwest and Southwest regions, respectively. Therefore, increasing solar power generation in a region not only helps in the reduction of CO_2_ emissions from the electricity sector within the region but also has an impact on the neighboring regions. Figure 6 shows the change in CO_2_ generation in the importing region corresponding to a 5, 10, 15, and 20% increase in solar generation in the generating region. The details and results of the interregional analysis, along with sensitivity analyses, are provided in Section B of the Supplementary Materials. From the interregional analyses, we conclude that it is important to account for interregional effects arising from the import and export of electricity. An increase in solar generation not only contributes to CO_2_ reductions within the generating region but also offers additional benefits by lowering emissions in neighboring regions that receive exported electricity. This interconnected dynamic highlights the broader systemic impact of increasing solar generation on reducing emissions across multiple regions.

## DISCUSSION

This study estimates the reduction in CO_2_ emissions associated with the increase in solar power generation on the hourly scale that accounts for immediate and time-lagged effects, by tailoring a distributed lag statistical model. In particular, our analysis identified an immediate and delayed effect of solar increase on CO_2_ reduction. Furthermore, our study identified regional variations in the impact of increasing solar generation on the change in CO_2_ emissions, with regions of California, Florida, Mid-Atlantic, Midwest, Southwest, and Texas demonstrating substantial reductions in CO_2_ emissions as a result of increased solar generation, while Central, New England, and Tennessee exhibited minimal effects. These differences between regions may be due to the general low solar generation in some regions, resulting in smaller absolute increases in solar even with a 20% increase, consequently resulting in less pronounced changes in CO_2_ emissions. In addition, factors such as geographical limitations or economic considerations might limit the adoption of alternative energy sources, further influencing the observed variations between regions. Our findings underscore the importance of considering regional dynamics when evaluating the potential benefits of solar power adoption for CO_2_ emission reduction. We further estimated the annual change in CO_2_ emissions on increasing solar generation by 5, 10, 15, and 20%. Although, theoretically, a 15% increase in solar generation could lead to a maximum annual CO_2_ reduction (marginal emissions) of 21.07 MMT across all regions, our models estimate an annual reduction of 8.54 MMT. We provide more details on the computation of the theoretical maximum annual CO_2_ reductions in table S2. Note that our data-driven model directly estimates how CO_2_ emissions respond to increases in solar power in the real world and does not assume proportional displacement of other sources depending on the energy mix.

We also quantified the interregional implications of the increase in solar power, demonstrating the potential for CO_2_ emissions reduction in neighboring regions importing electricity. For example, an increase in 15% solar power generation in California leads to an estimated decrease of 913 metric tons of CO_2_ emissions in the Northwest per day. This analysis suggests the potential for cross-regional collaboration in adopting solar power as a means of collectively addressing CO_2_ emission reduction goals in a more integrated and effective way. For example, the United States would benefit more from solar power in general, if solar power can be developed in states with higher solar radiation potential and lower cost and solar generation can be delivered to regions with higher CO_2_ emissions. For our study, we consider models that account for the lagged effects up to 12 hours. Utility-scale photovoltaic storage systems typically offer durations of 4 to 12 hours, depending on factors such as round-trip efficiency and depth of discharge (*30*, *31*). We conducted sensitivity analyses to evaluate the impact of varying the lag durations (details provided in section SB). These analyses highlight the issues corresponding to assuming very short or excessively long lag durations. On the basis of these findings, models that allow up to a 12-hour delay seem appropriate. As storage capacities continue to grow in the future, longer lag durations are expected to offer more accurate and comprehensive information by better capturing delayed effects on CO_2_ reductions.

### Limitations of the study

While our model facilitates the estimation of hourly lag effects and nonlinear associations, several limitations should be noted. First, we assume that the hourly demand has a linear relationship with hourly CO_2_ emissions and the effects of solar generation on CO_2_ reduction in each region do not vary at different levels of demand (i.e., that the distribution of power generation sources remains, on average, similar across multiple levels of demand). A shift in the distribution of energy sources, where other renewable sources offset future increases in demand, can result in a different relationship between solar generation and CO_2_. These assumptions have several implications on our inferences and projections (see the “Statistical framework” section). Specifically, fluctuations in demand, climate, and policies in subsequent years may affect the solar-CO_2_ relationship. Similarly, the model estimates are likely to be biased when projecting beyond the maximum observed solar generation, limiting us to consider at most an increase of 20% in solar power generation. Second, when developing projections for CO_2_ reductions, we chose percentage increases relative to the 2022 median of hourly solar generation; selecting a different base solar level would likely produce varied estimates. We chose to evaluate uniform percentage increases in solar generation across regions for simplicity and improved interpretability for regional policymakers. However, the projected effects thus are largely driven by the differences in the baseline solar capacity across regions, although our region-specific model also captured the different electricity grids and solar power potentials. Third, estimates from the Carolinas, Mid-Atlantic, and Southeast regions should be interpreted with caution due to the indication of lack of convergence in these models. Furthermore, convergence was lacking in the interregional estimates for certain pairs of import-generating regions, such as Carolinas-Southeast, Mid-Atlantic–Midwest, Midwest-Tennessee, Northwest-Southwest, and Southeast-Carolinas. As a result, these pairs were excluded from interregional estimates. Last, there is the potential for unmeasured confounding due to the lack of information on incentives governing the utilization of alternative renewable sources for electricity generation and regulatory frameworks affecting fossil fuel–fired power plants in various geographical regions.

### Strengths

This study provides a comprehensive understanding of the potential impact of increasing solar generation on reducing CO_2_ emissions generated by the US electricity sector. We address a notable gap in the existing literature by providing a data-driven analysis to estimate the effects of increases in solar generation on CO_2_ emissions reduction, accounting for the possibility that there may be immediate and delayed effects due to storage technologies or other incentives for the production of clean energy. This study also recognizes the importance of considering interregional electricity exchanges and quantifies the impact of solar generation on reductions in CO_2_ emissions in neighboring regions. The comprehensive dataset that we have curated, which spans more than 5 years, is publicly available.

### Policy implications

Our findings highlight the importance of implementing policies that encourage the adoption and investment of solar infrastructure to achieve the CO_2_ emission reduction goals of the US electricity sector, as established by the IEA (net zero emissions from the US electric sector by 2050) and the EPA (1380 MMT CO_2_ reductions by 2042). A recent study by Climate Central (*32*) reveals a notable increase in solar power production in the United States, with an estimated 238,121 gigawatt-hours generated from utility and small-scale solar installations in 2023, marking a 16% increase from 2022. Furthermore, the IEA has emphasized the imperative of expanding solar generating capacity, highlighting plans to add 630 gigawatts of solar photovoltaics to the energy mix (*12*). In our study, we complement these efforts by evaluating the alignment between solar energy expansion and CO_2_ emissions reduction targets, and, in particular, we estimate how much solar power generation needs to be further expanded to effectively meet established emission reduction targets. To enable substantial expansions of solar energy, a continued decline in the cost of modules and installations, which was closely related to the global supply chain (*33*), and policy incentives such as the federal investment tax credit, would be essential to encourage the deployment and utilization of solar energy systems for residential and commercial use (*34*). Our findings on the interregional effect align with the growing emphasis on solar power adoption; for instance, the installation of a giant battery in California helps to stabilize the grid across neighboring regions while also allowing for continued solar power during nighttime hours (*35*). Consequently, our study emphasizes the importance of improving interregional transmission capacity and exploring opportunities for collaboration between regions to maximize the benefits of emission reduction, which is in line with the latest efforts.

## MATERIALS AND METHODS

### Hourly electricity generation and CO_2_ emissions datasets

Our datasets (*36*) are curated from the EIA for 13 distinct regions: California, Carolinas, Central, Florida, Mid-Atlantic, Midwest, New England, New York, Northwest, Southeast, Southwest, Tennessee, and Texas between 1 July 2018 and 30 June 2023. Each regional dataset offers a consolidated view of electric power generation, total energy demand, and CO_2_ emissions during each hour at the regional level. The datasets provide insights into the electricity generated per hour (measured in megawatt-hours) obtained from each source, including coal, natural gas, nuclear, solar, wind, and hydro. Furthermore, the EIA datasets include estimations of hourly CO_2_ emissions originating from electric-generating units by using the hourly net generation of the electric-generating units and average CO_2_ emission factors (*29*). For additional information on the datasets, please refer to the official webpage of the EIA’s Grid Monitor, available at https://eia.gov/electricity/gridmonitor/about. Additional information and documentation are available at https://eia.gov/electricity/data/state/.

### Data cleaning and preprocessing

To use the EIA datasets for estimating the relationships between hourly solar generation and CO_2_ emissions, we took the following steps:

1) The New York region was removed from our analyses as the EIA dataset contained no information regarding solar generation in this region.

2) The information on the hourly solar energy generation and estimated CO_2_ emissions is available beginning 1 July 2018. Thus, we extracted the data between 1 July 2018 and 30 June 2023 for our analysis.

3) Negative and “NA” solar generation entries were replaced with zero or removed, respectively. Negative values occurred in several regions and almost entirely between the hours of 1700 and 0700 (i.e., nighttime). Approximately 10% of the solar data were changed from negative to zero and missingness of solar generation data occurred in 0.4% of rows.

4) Furthermore, we identified 17 rows in the dataset corresponding to the New England region, where the total electricity generation from various sources and CO_2_ emissions were reported as zero. We considered these instances as invalid data points and consequently eliminated those from our analysis to avoid misleading interpretations.

### Statistical framework

We develop a modeling framework to estimate the potential decrease in CO_2_ emissions associated with potential increases in solar power generated within a region, using the EIA datasets. Our model relies on the following assumptions. First, the relationship between solar power and CO_2_ is monotone—as solar power increases, the CO_2_ emissions will decrease or remain the same. Second, increased solar power allows for decreased energy generation from fossil fuel sources. The amount of CO_2_ that is offset by increased solar power generation depends on the existing fossil fuel energy source, each of which has a different CO_2_ emission factor. For instance, increasing solar power generation in a region heavily reliant on coal-fired power plants, emitting approximately 1 metric ton of CO_2_ per MWh of electricity generated, would yield a substantial CO_2_ offset compared to a region predominantly relying on natural gas, which emits about 0.4 metric tons of CO_2_ per MWh of electricity generated. Hence, we assume that the relationship between solar power generation and CO_2_ emissions is not strictly linear. As an example, small amounts of solar may first reduce electricity generation from the more flexible natural gas power plants, decreasing CO_2_ emissions by a factor related to the fuel source; as solar generation increases, coal power generation sources may also be decreased, and these sources have a different CO_2_ emission factor. More details on model selection and sensitivity is deferred to section SA. The final assumption we make on the relationship between solar energy and CO_2_ emissions is the potential for time-lagged effects of solar. Because of known patterns in solar generation (the sun comes up every morning and goes down every evening), as well as related changes in demand, energy pricing, and potential for energy storage, the amount of solar produced in a given hour may lead to a reduction in CO_2_ emissions both immediately or at some point in the future. To eliminate the issues corresponding to very short or excessively long lag duration (see section SB), we consider a maximum 12 hours of lag time by which solar may be related to changes in CO_2_ emissions.

To accommodate our modeling assumptions, we fit a nonlinear distributed lag model to estimate the relationships between CO_2_ emissions, denoted as *y_rt_* in region *r* during hour *t*, and solar energy generation during the current and past *L* hours denoted as $x_{\mathrm{rt}}={\left[x_{\mathrm{rt}},\ldots ,x_{r\left(t-L\right)}\right]}^{\prime }$ ; we set *L* = 12. Furthermore, we account for the current energy demand, *z_rt_*, as well as a smooth effect of time considering daily, seasonal, and yearly variations. We consider an additive relationship under a Gaussian model

$$E\left(y_{\mathrm{rt}}\right)=f\left(x_{\mathrm{rt}}\right)+h\left(t,r\right)+z_{\mathrm{rt}}\gamma_{r}$$ (1)

where $f\left(x_{\mathrm{rt}}\right)=\sum_{l=0}^{L}w_{r}\left(x_{r\left(t-l\right)},l\right)$ and $w_{r}\left(x,l\right)$ is a distributed lag nonlinear function (*27*, *28*), h(t,r) is a smooth function of time, and $\gamma_{r}$ is a coefficient defining a linear relationship between demand and total CO_2_. The smooth function of time, h(t,r) , is parameterized in two parts: first by a spline basis transformation on date with 10 degrees of freedom, and second by a spline basis transformation on hour of day with 5 degrees of freedom. The statistical model generates a smooth and nonlinear relationship between hourly CO_2_ emissions from the grid and the solar power generated during the current hour, as well as each of the past 12 hours, while controlling for the demand and other parameters (detailed in the “Inference” section). We aim to estimate changes in marginal emissions and thus use the relationship to estimate the change in CO_2_ emissions associated with additional hourly solar power generation. To account for regional differences, we fit the model separately for each power generation region.

### Inference

Our interest lies in how increases in solar power generation are related to changes in CO_2_ emissions (metric tons). To better represent the typical solar generation and avoid extrapolating the results from our model, we consider the median solar generation at each hour of the day during the year 2022 as a baseline for comparison. We then define four counterfactual increases in solar generation (5, 10, 15, and 20%), which we calculate as a proportional increase to the median hourly solar generation. For hour h∈{0,…,23} , let $x_{\mathrm{rh}0}$ equal the median solar generation and $x_{\mathrm{rhp}}=\left(100+p\right)\%\cdot x_{\mathrm{rh}0}$ equal solar generation after a (100 + *p*)% increase. On the basis of estimates from Eq. 1, $w\left(x_{\mathrm{rhp}},l\right)-w\left(x_{\mathrm{rh}0},l\right)$ equals the change in CO_2_ emissions due to increase in solar power generation l∈{0,…,L} hours ago. Similarly, $\sum_{l=0}^{L}w\left(x_{r\left(h-l|24\right)p},l\right)-w\left(x_{r\left(h-l|24\right)0},l\right)$ , where (h−l∣24)=h−lmod24 equals the total change in CO_2_ emissions at hour *h* for a *p*% change in median solar generation during the current and all previous hours. Last, to estimate the change in CO_2_ resulting from a *p*% increase it median solar generation during an entire day, we calculate

$$\sum_{h=0}^{23}\sum_{l=0}^{L}w\left(x_{r\left(h-l|24\right)p},l\right)-w\left(x_{r\left(h-l|24\right)0},l\right)$$ (2)

These estimates are then multiplied by 365 to estimate the total annual CO_2_ reductions on *p*% increase at each hour of the day over the year. Posterior samples from the Markov Chain Monte Carlo (MCMC) method were used to compute the median change in CO_2_ emissions and construct 95% credible intervals.
