# Supplementary material for: Quantifying effects of solar power adoption on CO2 emissions reduction
Source: Sci Adv. 2025 Jul 30;11(31):eadq5660. doi: 10.1126/sciadv.adq5660 (PMC12309663; doi:10.1126/sciadv.adq5660)
Supplement: Supplementary file 1 — Supplementary Text Figs. S1 to S6 Tables S1 and S2 [file sciadv.adq5660_sm.pdf]

Science Advances
AAAS

Supplementary Materials for

Quantifying effects of solar power adoption on CO_2 emissions reduction

Arpita Biswas *et al.*

Corresponding author: Francesca Dominici, fdominic@hsph.harvard.edu

Sci. Adv. **11**, eadq5660 (2025)
DOI: 10.1126/sciadv.adq5660

This PDF file includes:

Supplementary Text
Figs. S1 to S6
Tables S1 and S2

A Model Selection and Sensitivity

To estimate $w_r(x, l)$ we employ a regression tree approach for estimating distributed lag nonlinear models (TDLNM) incorporating monotonicity constraints (*26*). This data-driven approach has two key advantages over traditional modeling approaches. First, the constrained distributed lag model produces lower variance estimates of lagged effects when repeated measurements of solar generation are taken at high temporal resolution and highly correlated. As a sensitivity analysis, we compare the TDLNM to an unconstrained DLM. fig. S2 showcases distributed lag estimates for a single region comparing TDLNM and an unconstrained distributed lag model indicating the potential biases of not accounting for highly correlated solar generation measurements. Second, TDLNM allows for the inclusion of monotonicity in $w_r(x, l)$ at each lag time l. A non-monotonic, nonlinear estimate of the relationship between solar generation and CO_2 often results in excessive 'wiggliness', as shown in our sensitivity analysis in fig. S3, which is counter to our assumption that increasing solar will not increase CO_2 emissions. We follow the work by Mork and Wilson (*26*) to select hyperparameters for fitting TDLNM.

For a Bayesian model that is estimated using Markov chain Monte Carlo (MCMC) procedures, our primary concern is the convergence of the parameters. Each model is fit using 5 independent Markov chains, each chain with 2,000 warm-up samples followed by 5,000 samples thinned by a factor of 10 to reduce autocorrelation between samples. The lowest median Gelman-Rubin $\hat{R}$-statistic was used to decide the number of regression trees in the model (20, 40, or 80) and traceplots were further utilized to assess convergence (fig. S4). We note that Carolinas, Mid-Atlantic, and Southeast regions displayed evidence of multimodality in the traceplots leading to increased uncertainty and lower confidence regarding the estimates of the solar-CO_2 relationship.

B Sensitivity Analyses Considering Different Lag Hours

We conducted experiments to showcase the sensitivity of the estimations to the number of lag hours considered while generating the model. For illustrative purposes, we present results derived from models trained on California data. For the analyses, different numbers of lag hours are considered to generate separate models. fig. S5 displays the estimated cumulative change in CO_2 emissions at

each hour for a 15% increase in solar power during each of the previous $x = \{4, 8, 12, 16\}$ hours, where x is the number of lag hours considered while training the model. The analysis reveals that the median estimated CO_2 reduction is not substantially different between the models. However, here are some interesting insights that highlight the issues with considering too few or too many lag hours while generating the model.

- The 4-hour lag model estimates almost zero CO_2 reductions during nighttime, whereas the 8-hour, 12-hour, and 16-hour lag models indicate non-zero reductions. This is consistent with our expectations, since the 4-hour model cannot account for delayed CO_2 offsets that occur after sunset.
- The estimated reduction in CO_2 is slightly lower for the 4-hour and 8-hour models compared to the 12-hour and 16-hour lagged models. This suggests that a low number of lag hours is insufficient to capture the complete CO_2 offset, indicating the necessity of longer lag periods.
- We also observe that the variance increases when the number of lag hours is higher (for example, see the 16-hour lagged model), which may arise from greater temporal correlations in the 16-hour lag model compared to the models that consider lower lag hours.

C Daily Estimates

The table S1 shows the estimated daily cumulative change in CO_2 emissions along with their 95% credible intervals, corresponding to increases of 5, 10, 15, and 20% in solar power generation across each region. This table serves as a detailed quantitative representation of Fig. 4 in the main text illustrating the same estimations in the form of a bar chart.

D Interregional Effects

We adopt the same method—nonlinear distributed lag model—to estimate the relationships between CO_2 emissions, denoted as y_{rt} in region r during hour t, and solar energy generation of a neighboring region r' during the current and past $L = 12$ hours denoted as $\mathbf{x}_{r't} = [x_{r't}, \dots, x_{n(t-L)}]'$. Furthermore, we account for the current electricity demand of region r, z_{rt}, solar generated in the

region r, x_{rt}, as well as a smooth effect of time considering daily, seasonal, and yearly variations. We consider an additive relationship under a Gaussian model,

$$E(y_{rt}) = f(\mathbf{x}_{r't}) + h(t, r) + z_{rt}\gamma_r + s_{rt}\beta_r, \tag{S1}$$

All the notations have the same meaning and assumptions as explained in Section 4.2. To account for the interregional differences we fit the model separately for each tuple of neighboring regions—importing regions as r and exporting region as r'. Similar to the analysis within the same region, for the interregional analysis, we infer from the trained models the hourly change in CO_2 emissions associated with an increase in solar in a neighboring region from which it imports energy. We conducted experiments to showcase the sensitivity of the estimations to the number of lag hours considered while generating the interregional models. For illustrative purposes, in fig. S6, we present results derived from models that estimate the effect of increases in solar generation in California to the change in CO_2 emissions in California.

E Theoretical Maximum Annual CO_2 Reductions

The table S2 presents the annual increase in solar generation (in TWh = 10^6 MWh) corresponding to a 15% increase in solar generation in each region, alongside regional CO_2 emission factors for coal, natural gas, and petroleum (in metric tons per MWh) using data from the year 2022. The table also shows the theoretically maximum annual CO_2 emission reductions if the increased solar was completely displaced by coal, natural gas, and petroleum—these values are computed by multiplying the increased solar generation values in TWh and CO_2 emission factors. Additionally, we include our model's estimated total annual CO_2 emission reduction to highlight the gap between the theoretical maximum and our estimated reductions.

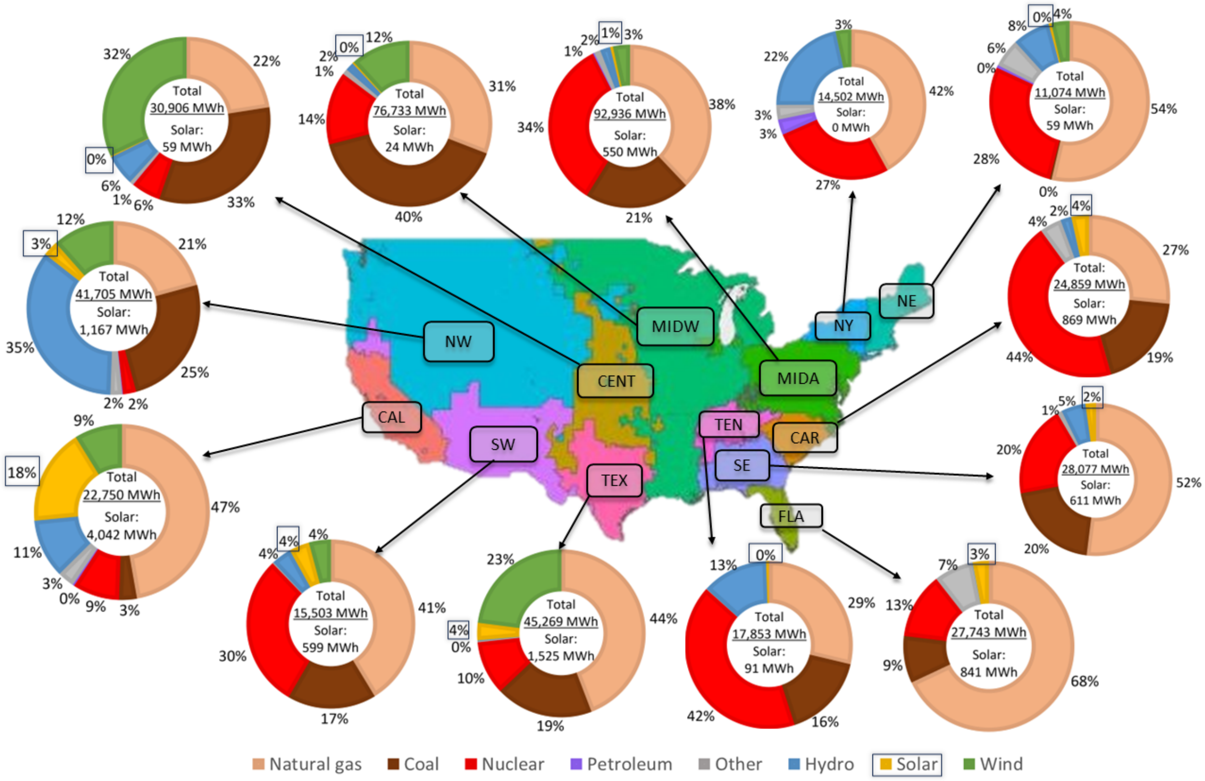

Figure S1: **Average hourly electricity demand (in MWh) and average hourly solar power generation (in MWh).** The average is taken across all hours during the period July 1, 2018, and June 30, 2023, across 13 geographical regions in the U.S. Additionally, color codes represent the proportional contribution of each source to the total electricity demand (as percentage). These metrics are derived through the aggregation of U.S. Energy Information Administration (EIA) data.

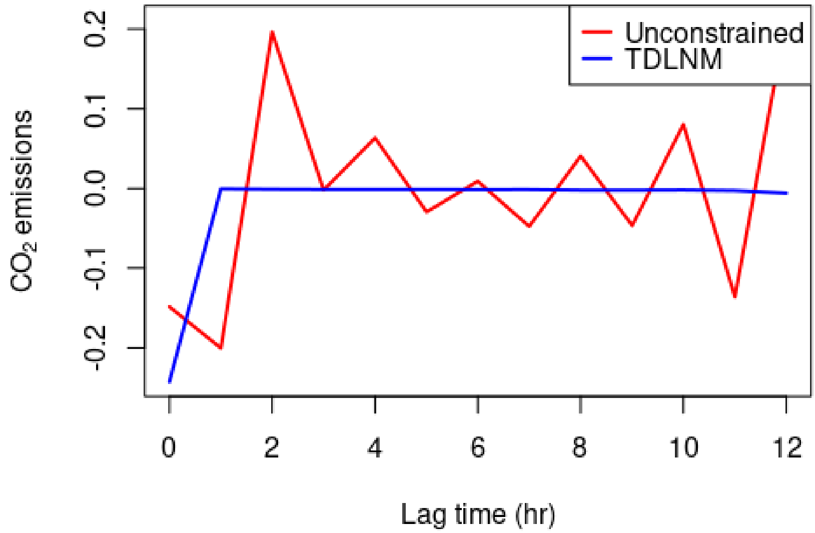

Figure S2: **Sensitivity to monotonicity constraints for the Texas power region.** TDLNM and unconstrained distributed lag models for unit increase in solar generation.

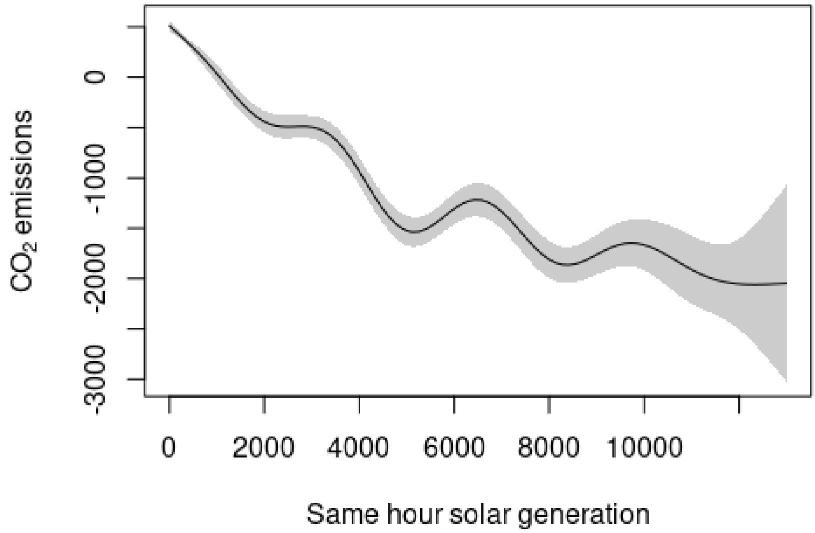

Figure S3: Same hour solar-CO_2 relationship without monotone constraint.

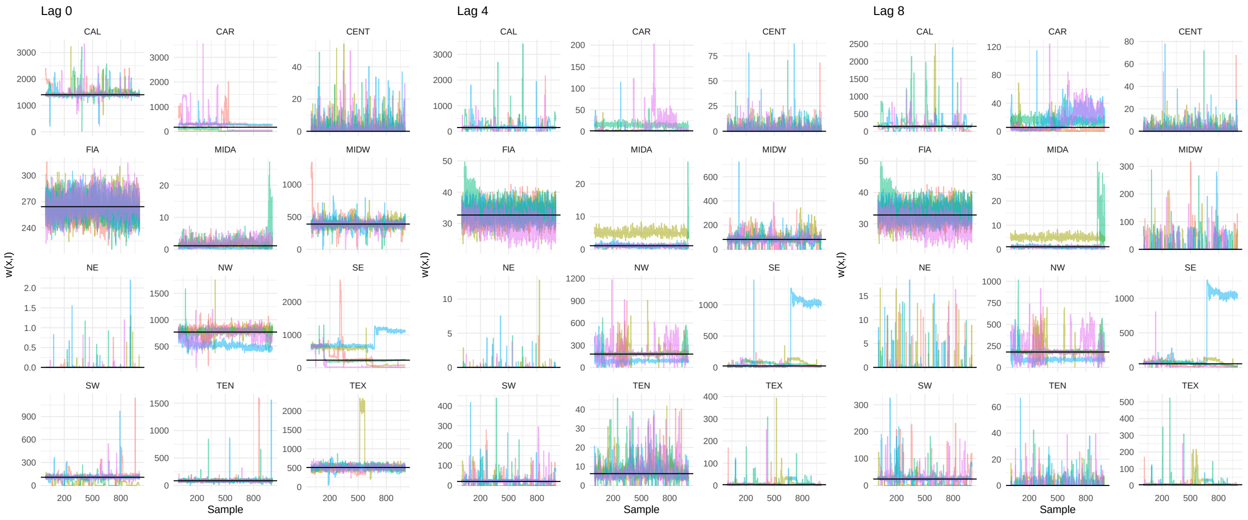

Figure S4: **Traceplots for each region at lags 0, 4, and 8 showing the estimated change in CO_2 at median solar generation**. Colors represent the independent Markov chains and the horizontal black line indicates the posterior median.

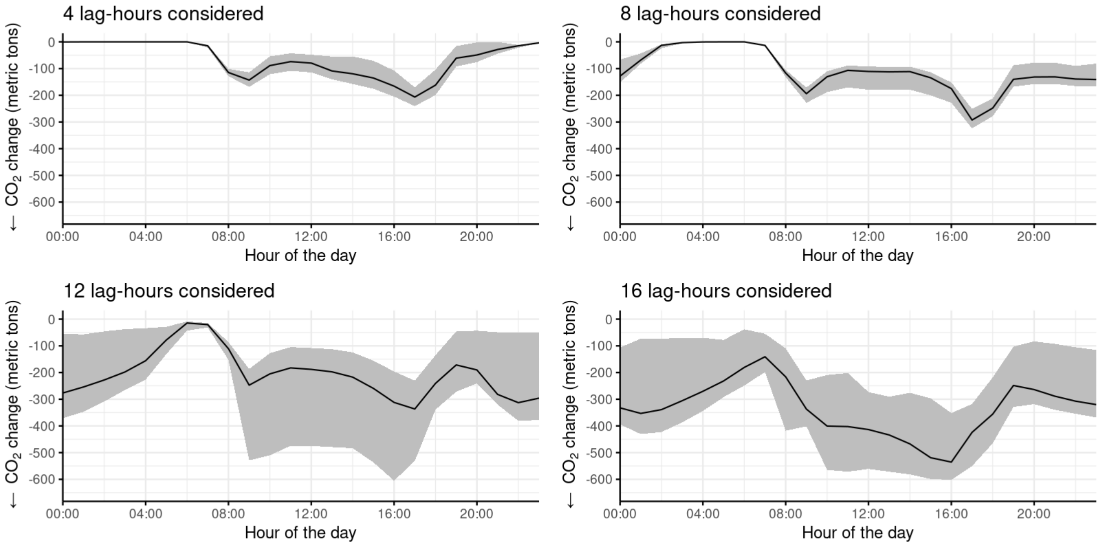

Figure S5: **Sensitivity to the number of lag hours considered in the regional models**. Each subfigure shows the estimated hourly change in CO_2 emissions in California for a 15% increase in solar power during each of the past x hours, where x is the corresponding number of lag hours considered. The analysis reveals that the median estimated CO_2 reduction is not substantially different between the models.

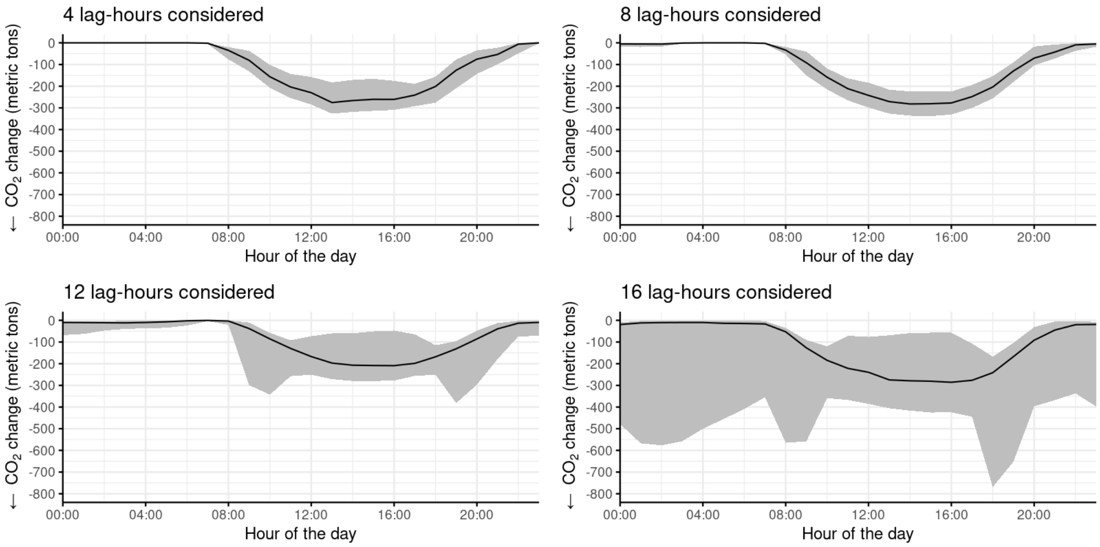

Figure S6: **Sensitivity to the number of lag hours considered in the interregional models**. Each subfigure shows the estimated hourly change in CO_2 emissions in Southwest for a 15% increase in solar power in California during each of the past x hours, where x is the corresponding number of lag hours considered. The analysis reveals that the median estimated CO_2 reduction is not substantially different between the models.

Table S1: **Estimated median change in metric tons CO_2 emissions (95% credible interval) per day from a region for a 5, 10, 15, 20% increase over the median solar generation (relative to 2022) within the same region.**

	**Percentage increase in solar generation**			
	5%	10%	15%	20%
California	-1554.3 (-2822.8, -1157.2)	-3020.5 (-5477, -2189.5)	-4357 (-7860.1, -3115.6)	-5538.7 (-9961.8, -3901)
Carolinas	-548.2 (-1258.1, -400.8)	-1011.8 (-2407.9, -763.2)	-1413.1 (-3436.9, -1084.5)	-1770 (-4332, -1365.6)
Central	-2.3 (-285.6, 0)	-4.4 (-613.5, 0)	-6.3 (-975.1, 0)	-8 (-1354.2, 0)
Florida	-649.6 (-681.3, -618.5)	-1251.4 (-1315.2, -1189)	-1791.8 (-1886.8, -1698)	-2265.1 (-2387.7, -2143.1)
Mid-Atlantic	-1562.3 (-5170.9, -1294.4)	-3011.1 (-9994.2, -2472.8)	-4317.5 (-14312.9, -3518.1)	-5474.8 (-18050, -4425.2)
Midwest	-939.7 (-2182.6, -582.1)	-1817.6 (-4279.2, -1124.3)	-2604.4 (-6226.1, -1601.6)	-3292.6 (-7823.2, -2017.9)
New England	0 (-1.5, 0)	0 (-2.8, 0)	0 (-4, 0)	0 (-5.1, 0)
Northwest	-446.7 (-1371.3, -365.4)	-828.1 (-2520.9, -680.3)	-1159.4 (-3470.9, -955.4)	-1448.3 (-4260, -1201.7)
Southeast	-654.6 (-1047.1, -469.6)	-1220.6 (-1965.8, -868.7)	-1717.8 (-2747.9, -1207.4)	-2167.1 (-3467.2, -1499.2)
Southwest	-1178.7 (-7153.4, -1069.9)	-2424.2 (-15113.7, -2183.5)	-3689.9 (-23523.4, -3305.8)	-4932.5 (-32022.6, -4382.2)
Texas	-785.1 (-1008.9, -568.8)	-1483.9 (-1953.5, -1041.7)	-2103.2 (-2823.9, -1436.4)	-2645.2 (-3624.7, -1779.3)
Tennessee	-95.6 (-130, -69)	-177.4 (-242.1, -127.7)	-247.3 (-340.3, -178)	-307.4 (-423.4, -221.5)

Table S2: **The gap between the theoretical maximum annual CO_2 emission reductions and the estimates we obtain**. The first column shows the regions and the second column shows the annual increase in solar generation (in TWh = 10^6 MWh) in each region corresponding to a 15% increase in solar generation in each region. The third, fifth, and seventh columns indicate the regional CO_2 emission factors for coal, natural gas, and petroleum (in metric tons per MWh) computed using data from the year 2022. The fourth, sixth, and eighth column shows the theoretically maximum annual CO_2 emission reductions if the increased solar was completely displaced by coal, natural gas, and petroleum, respectively. The ninth column provides the annual estimates we obtain. The last row of the table provides the total of each column.

**Regions**	**Increased solar (TWh)**	**Coal**		**Natural gas**		**Petroleum**		**Estimated CO_2 reduction (MMT)**
		**factor (MMT/TWh)**	**reduction (MMT)**	**factor (MMT/TWh)**	**reduction (MMT)**	**factor (MMT/TWh)**	**reduction (MMT)**	
**California**	6.376076	0.968158	6.173047	0.407998	2.601429	0.942476	6.009298	1.590304
**Carolinas**	1.459635	0.953332	1.391517	0.428644	0.625664	0.286314	0.417914	0.515768
**Central**	0.08322	1.06127	0.088319	0.48187	0.040101	0.941414	0.078344	0.00229
**Florida**	1.685643	1.013712	1.708757	0.39119	0.659406	0.942172	1.588166	0.654002
**Mid-Atlantic**	1.58947	1.009762	1.604986	0.389295	0.618773	0.871614	1.385404	1.575898
**MidWest**	0.969828	1.022923	0.992059	0.43165	0.418626	NA	NA	0.950592
**New England**	0.139613	1.029575	0.143741	0.39209	0.054741	0.940335	0.131282	0.00
**Northwest**	2.196132	1.034242	2.271333	0.426178	0.935944	0.938576	2.061237	0.423187
**Southeast**	1.228426	0.996818	1.224517	0.407778	0.500925	0.946008	1.162101	0.627008
**Southwest**	1.37058	1.01337	1.388904	0.419391	0.574809	0.93853	1.286330	1.346809
**Tennessee**	0.207019	1.09281	0.226232	0.393826	0.081529	0.950796	0.196832	0.090266
**Texas**	3.644543	1.05799	3.85589	0.414435	1.510427	NA	NA	0.767663
**Total**	20.950183		**21.0693**		**8.622373**		**14.31691**	**8.5438**
